# Towards a Model of Teacher Well-Being from a Positive Emotions Perspective

**DOI:** 10.3390/ejihpe10010035

**Published:** 2020-02-21

**Authors:** Loredana Manasia, Andrei Pârvan, Melania Macovei

**Affiliations:** 1 Department of Teacher Education and Social Sciences, University POLITEHNICA of Bucharest, 060042 Bucharest, Romania; melania.macovei@upb.ro; 2 Department of Sciences of Education, University of Bucharest, 050663 Bucharest, Romania; andrei.parvan@unibuc.ro

**Keywords:** teacher well-being, teaching emotions, happiness, enjoyment of teaching, teaching anxiety

## Abstract

Teacher well-being represents a key factor in assuring the quality of learning in terms of both process and outcomes. Despite a growing literature addressing the role of job demands and job resources in teacher well-being, fewer studies have focused on the effect of individual variables. The present paper aims at developing a teacher well-being model using self-efficacy and teaching emotions such as enjoyment of teaching, anger and anxiety to explain the influence of job demands and job resources on teachers’ subjective happiness. A cross-sectional quantitative design was applied to a sample of 1092 Romanian pre-university teachers. The participants completed a self-report questionnaire. Descriptive statistics, factor analysis and structural equations modelling were used to analyse the data. The findings indicate significant paths between the variables included in the model. Thus, job resources have a considerable positive influence on the enjoyment of teaching and the teachers’ subjective happiness, having a more powerful effect than personal resources, namely self-efficacy. In turn, perceived self-efficacy mediates the effect of job demands on teaching emotions and subjective well-being. It is argued that the enjoyment of teaching has a notable effect on teachers’ general well-being.

## 1. Introduction

In recent years, there has been a steady change in well-being research, as Capone and Petrillo suggest [1]. This change led to a shift from the research of distress symptoms to the investigation of personal strengths, resources and well-being from a positive psychology perspective [1,2,3]. In the same line of thought, Diener [4] puts forward a broader definition of well-being, understood as the complex result of people’s cognitive and affective evaluations of their lives. This includes a number of separate components: life satisfaction, satisfaction with important domains, positive affect and low levels of negative affect. The approach to defining a good life was called subjective well-being or happiness [4,5,6]. Within the framework of the theory of maximal development of human potential, Ryan and Deci [7] define well-being as an open, healthy operation based on the subject’s commitment. In their view, ‘developing the individual potential favours the well-being, but does not grant it’ [7]. 

A large empirical literature suggests that well-being is a multidimensional construct [4,8,9,10,11,12] and can be defined by the prevalence of the positive, not just the absence of the negative, involving both optimal experience and positive functioning [13,14]. Keyes [15] advocates that a comprehensive approach of well-being would include both hedonic and eudaimonic approaches. Consistently, Huppert [13] and Kern [9] emphasize the need to relate both feeling good and functioning well in theoretical approaches of well-being. Dodge and her collaborators raise criticism of the omnibus nature of previous approaches to defining well-being [16]. Consequently, they build upon the dynamic equilibrium theory and point forward to a new definition of well-being encompassing three key elements: a set-point for well-being, the equilibrium and the balance between psychological, social and physical challenges and resources. The systemic functioning of the three elements is explained by Kloep et al.: ‘Each time an individual meets a challenge, the system of challenges and resources comes into a state of imbalance, as the individual is forced to adapt his or her resources to meet this particular challenge’ [17]. Well-being is a balance point between support, resources, and autonomy with challenges, demands, and intensity, argue Wassell and Dodge [12]. When challenges outrun the resources, a state of tension occurs, destabilizing the ‘balance’. To increase well-being, it is important to identify whether more demand and challenge are needed or whether the individuals need more support, autonomy or feedback to reach an equilibrium state. Essentially, well-being state is reached when the subjects hold the psychological, physical, and social resources needed in order to optimally meet a challenge in the environment. 

The literature points out several negative effects of an imbalanced relationship between challenges and resources within the teaching profession. The teacher attrition phenomenon is a recurring problem on the agenda of the worldwide educational systems. Recent research suggests that approximately 40% of teachers abandon the profession less than five years after the teaching career onset [18,19]. Likewise, the National Association of Schoolmasters Union of Women Teachers (NASUWT) Big Question Survey conducted in 2017 in England reveals that 61% of teachers were thinking of quitting the profession [20]. The costs related to teacher attrition are significant and they draw negative effects: discontinuity of study programs and the need to invest in training new teachers and implementing employability programs for them [21]. Some of the early-career teachers experience pressure, conflictual situations and stress [22], leading to low levels of motivation and involvement in the teaching activity [19,23,24]. Other authors highlight the implications of burnout syndrome, pointing out its extensive dimension with recurrent absenteeism and high costs for healthcare [25,26,27]. Although an extensive corpus of research investigates stress and burnout in teachers, fewer studies focus on emotions and well-being. 

Within the framework of the dynamic equilibrium theory proposed by Dodge and her collaborators, the main purpose of this research study was to propose a statistical model of teacher well-being, postulating relationships between job resources, job demands, perceived self-efficacy, teaching emotions and subjective happiness, seen as a dimension of well-being. The following sections discuss the variables in the model and their relationship with teacher well-being. 

## 2. Challenges and Job Demands in Teacher Well-Being

Teachers constantly find themselves in contexts where they have to provide optimal answers to the demands of the educational environment and of the students [28]. Bakker and Demerouti [29] state that job demands ‘refer to those physical, psychological, social, or organisational aspects of the job that require sustained physical and/or psychological (cognitive and emotional) effort or skills and are therefore associated with certain physiological and/or psychological costs’. Skaavik and Skaavik [30] indicate that the concepts of job demands and job stressors could be used interchangeably. 

In explaining the job demands category, the literature includes the subcategories of physical, cognitive and emotional demands. Skaavik and Skaavin [30] suggest that time pressure (or work overload), discipline problems (or student disruptive behaviour), low student motivation, large student diversity, conflicts with colleagues, lack of administrative support, value conflicts, and role ambiguity are the most frequent and impactful job demands in schools. In recent years, there has been a rise in research studies reporting an increase in teacher workload [30]. Examining teacher job satisfaction, Cross [31] finds that teachers experience depression, anxiety and stress mostly due to real or perceived workload.

Yin, Huang and Wang [32] have studied the range of emotions associated to teaching and emotional regulation. The research results reveal contrasting models of interaction between the emotional demands of the teaching activities, trust among colleagues and the teachers’ well-being. Moreover, the study proves that an efficient strategy protecting teachers’ well-being is based on the full awareness of emotions and affective states typical of the school environment, the skilful management of emotional regulation strategies, and the aspects of building an emotional climate based on trust and co-participation. Other researchers report student misbehaviours and time pressure to be associated with low engagement and motivation, stress, and intention to leave the profession [5,30,33,34]. ‘Organisational and social pressures, such as the administrative work volume, the class management issues, and the lack of a supervisor and of the team support, were extensively studied’ [35,36,37]. Split et al. [38] approach the impact of the teacher–student relationship on teachers’ well-being. The teacher–student relationships, characterised by conflict and lack of trust, have harmful effects on learning outcomes [39]. Even so, little is known about the ‘interpersonal requirements experienced by teachers from their students’ [40]. In addition, it has been contended that there is a low awareness of the internal needs the teachers themselves might have for positive, personal relations with the students. In the present study, three job demands were researched, namely (1) disruptive behaviours of the students (e.g., disobedience, absenteeism); (2) overwork and insufficient time for carrying out the work tasks (e.g., a high number of work tasks and perception that their completion is impossible, such as preparing the lessons, correcting the tests, and others); (3) conflict situations (e.g., in relation to the parents or school leadership). 

## 3. Resources and Well-Being in Teachers 

Following an analysis conducted by Bakker and Demerouti [29], the resources can be defined as attributes of the working environment that are deemed to be mediators in the process of goal achievement and contribute to decreasing psychological and physical costs of job demands. In regard to resources, several categories have been suggested. Hence, there are organisational resources, interpersonal resources, or resources relating to specific work tasks [32]. Recently, research studies have started to consider personal resources in relation to burnout and engagement. As previous studies have suggested [5,30,32], most research focuses on teacher autonomy, positive relationships with fellow teachers, leadership, parents and students, feedback, and development opportunities as teachers’ job resources. Vera, Salanova, and Lorente [41] point out self-efficacy as one of the most powerful personal resources. 

In the context of the present study, five job resources and personal resources such as self-efficacy and teaching emotions were retained. Therefore, autonomy, value of teaching profession, feedback, professional and social positive relationships, and job variety were researched as job resources.

### 3.1. Job Resources

According to previous research findings [28,42,43,44], autonomy is a central dimension of teacher professionalism. The Organisation for Cooperation and Economic Development (OECD) [43] states that autonomy strongly relates to both decision making and empowerment and ‘it recognizes teachers’ capacity to make sound professional judgement’. Teacher autonomy can be discussed in relation to various domains, as Darling-Hammond [45] suggests, such as curricular design, instruction and assessment planning, and classroom management. Discussing the role of teacher autonomy, Wang et al. [44] conclude that this resource is strongly associated with high levels of professional responsibility and personal and professional growth. 

Another valuable job resource that has been investigated in the context of the present study is the value of the teaching profession, operationalised through social prestige, safety, and financial comfort. Although teachers play a crucial role in society, OECD [46] reports that less than one-third of the teachers believe that their profession is valued in society. The same study reveals a causal relationship between the performance of education systems and the degree to which the teaching profession is valued in society. Another dimension of the value of the teaching profession is the level of payment and, consequently, the financial comfort it supports. The NASUWT 2017 survey [20] has made it known that 79% of teachers declared they do not believe that teacher salaries are competitive when compared to other professions. The level of payment was associated with teacher attrition [20]. Hendricks [47] finds that a one percent increase in teacher pay reduces teacher turnover by 1.4%.

The relationships teachers have with their colleagues, their school leaders and their students are important determinants of teacher well-being [46,48]. Correspondingly, it has been argued that developing positive relationships with fellows and students is of significant impact on job satisfaction and self-efficacy [49,50]. Previous studies revealed that positive professional relationships with fellows and supervisors are associated with high levels of work engagement, strong motivation and low levels of stress and burnout [5,30,32,34,46]. In the same line of thought, Sato and Haegele [51], argue that formal and informal relationships and networking can positively impact teachers’ professional engagement. Narrowing the perspective to early-career teachers, actively supported mentoring activities and induction programmes are critical in supporting teacher professionalism [28,45,52]. As other studies point out, novice teachers are affected by a lack of peer support [53,54]. As Spilt, Koomen and Thijs [38] highlight, student–teacher relationships are also of great relevance in explaining teacher well-being. Interactions with students are deemed an important source of emotions for the teachers. The researchers introduced the concept of emotional labour to describe the emotional requirements inherent to the didactic profession [55]. Chang [16,56] discusses burnout and its related emotions in order to describe the emotional experiences of teachers when dealing with disruptive students, thus highlighting the importance of discrete emotions of teachers for their well-being (for instance, anger, frustration, anxiety and guilt). 

OECD [43,46] analysis suggests that feedback can have a significant effect on teachers’ instructional activities, their motivation and attitudes towards teaching, and learning outcomes alike. Although there is room for research regarding the impact of feedback in relation to its source, a body of studies has pointed out that peer feedback is generally regarded as useful for professional growth [26,46,57,58]. Salanova and Schaufeli [59] highlight that proper feedback fosters learning and, consequently, improves work performance. In addition, Danielson [60] depicts the receptivity to feedback from colleagues as an attribute of a true practitioner. The potential of job resources has also been endorsed by more traditional yet influential theories and models, such as Job Characteristics Theory (JCT) [61,62]. According to JCT, the motivational potential of the job is influenced by the presence of five job characteristics: task identity, skill variety, task significance, autonomy, and feedback. As reported by Salanova and Schaufeli [59], job variety can be identified as a contextual job resource. It can be understood as the accommodation of the use of various skills and talents (skill variety) to the variety of different activities (task variety) [63].

### 3.2. Personal Resources

#### 3.2.1. Perceived Self-Efficacy

The concept of self-efficacy refers to the perceptions and/or beliefs of a subject with regard to the individual’s abilities to efficiently carry out an activity or a sequence of activities [50,64,65]. Klassen and Chiu [50] identify a significant corpus of research papers endorsing the role self-efficacy played in reaching an optimum level of personal and professional success in various fields, such as education, health, sports or even businesses. Bandura [64] himself, in the article dedicated to self-efficacy conceptualisation, endorses such a hypothesis. Capone and Petrillon [1] summarise a body of research studies and conclude that self-efficacy is related to burnout, job satisfaction and mental well-being in teaching profession. Caprara et al. [49] found that self-efficacy is associated with high levels of happiness in teachers. 

The analysis of the definitional field dedicated to the self-efficacy concept indicates the presence of certain convergence areas between the opinions expressed by researchers and theoreticians. Thus, operational definitions might reflect the subjects’ conceptions about their own capacities, and they might be translated using the modal verb ‘could’ (what I could do, rather than what I shall do), say Klassen and Chiu [50]. Bandura [66] explains the fact that ‘could’ expresses a judgement of competency, a value expression from an individual perspective. 

As Katsantonis [65] suggests, self-efficacy is a multidimensional construct. Bandura [64] proposes a quadripartite structural model of self-efficacy, identifying the following pillars: enactive experience, vicarious experience, verbal persuasion and affective interpretation of psychological states. The subject’s self-efficacy operates as an intrapersonal motivating factor synthesising the essential aspects of the human being as action agent: effort and persistency in reaching individual or professional goals. For teachers, self-efficacy may contribute to increasing persistency in working with heightened personalities, as well as to potentiating enthusiasm and involvement in the teaching activity [67]. 

Klassen and Durken [67] argue that previous studies researching self-efficacy raised various conclusions, not necessarily convergent. Some research studies claim the existence of modest variations with the evolution of the teaching career [68], while others identified unequal evolution rhythms, describing a time of intense accumulations at the career inception (particularly during the initial training). Klassen and Durkesen [67] have conducted a study in which they tested the role of the student teaching practice on self-efficacy and professional stress. The conclusions drawn endorse the dynamic nature of self-efficacy, responding to the influence of certain exogenous variables. Klassen and Tze [67] argue that no variable could have an invariable effect on self-efficacy, being influenced by contextual factors, the validity of the research instruments and the reliability of the self-appraisals made by subjects. This approach is convergent with Bandura’s statement [64], which identified a strong associative relation between the professional success recorded in the early stages of the career and self-efficacy level, the hypothesis being that success contributes to the positive increase in self-efficacy. 

There is scientific evidence suggesting that teacher perceived self-efficacy influences student learning outcomes and boosts job satisfaction [69,70]. Skaalvik and Skaalvik [71] find a positive association between teachers’ self-efficacy and student achievement and motivation. Complementarily, self-efficacy tends to be positively associated with enthusiasm, commitment and job satisfaction [71]. Drawing on these findings, it is reasonable to assume that teachers’ self-efficacy will have an effect on the enjoyment of teaching and subjective well-being.

#### 3.2.2. Teaching Emotions

Emotions play a key-role in the research focused on stress and burnout [72,73]. Emotions’ ubiquity in the classroom is a reality per se, causing vitalising or asthenic effects on both categories of actors involved in the didactic process. Even though in the last two decades a large scientific literature on emotions has been developed, there is still a lack of consensus on the definition of emotions [74,75]. The work of Fredrickson and Joiner [76] laid the foundation for researching the impact of positive emotions on psychological well-being. The broaden-and-build theory of positive emotions states that positive emotions are evolved adaptations that contribute towards building lasting resources [77,78,79]. Even though positive emotions are few and rather diffuse, they have a strong mediating role on individual’s physical, intellectual, social, and psychological resources [76,78,80]. On balance, positive emotions may be a fundamental human strength central to the study of human flourishing, as Fredrickson argues [80]. Longitudinal field experiments show that individuals who experienced positive emotions gain increases in personal resources [81]. In turn, those gains in resources positively impact subjects’ life satisfaction Cohn and his collaborators [77] explain that happiness or subjective well-being was largely proven to be a precondition of positive life outcomes, such as financial success, supportive relationships, and mental health. 

In relation to teaching, Pekrun, Goetz, Titz and Perry [82] claim that positive emotions are associated with efficient problem solving with forms of secure attachment; they endorse self-regulation and influence group dynamics. Ashby et al. [83] state the same hypothesis and indicate a relationship between activating cognitive resources, the increased performance of the teaching process, and the presence of positive emotions. Moreover, all these lay the foundation of interests (as cognitive and motivational structures), boosting the personal interest of oneself for engaging in future educational experiences [84]. The existing studies provide evidence that teaching emotions are clearly related to teacher well-being, burnout and teacher attrition [56,85]. Moreover, they are highly likely to influence the dynamic of teacher–student relationships, which, in turn, are associated with distress and low levels of work engagement [85].

The present research is rooted in the model proposed by Frenzel, Pekrun, Goetz, Daniels, Durksen, Becker-Kurz and Klassen [85]. The model includes three emotions that the authors find relevant and impactful: enjoyment of teaching, anxiety, and anger. To theoretically fundament the occurrence of emotions as part of the teaching activity, the cited authors follow a setting tightly related to the compositional definition of emotions, namely, the appraisal theory [86]. The appraisal theory stipulates that an event itself does not generate emotions, but rather the appraisal made by the person experiencing the event. The theory proposes several dimensions from which the appraisal could be made: the event novelty, and its objective, congruency or controllability, which, conjugated, determine the intensity and the quality of the emotional reaction. As other studies have proved [85], enjoyment can be considered the most salient positive emotion. Enjoyment (joy [78] or happiness [85]) does not represent a unique affective state but a family of states, as Fredrickson [78] suggested, and shares a conceptual space with amusement and gladness. In turn, anger seems to be the most prominent negative emotion for teachers [74,85]. In relation to psychological well-being, anxiety has gained scientific interest due to its negative impact [74,75,87]. A contribution of the current research is to consider teaching emotions and, in particular, positive teaching emotions, as personal resources that could be boosted by contextually appropriate job resources and together influence the teachers’ subjective happiness.

**Figure 1:**
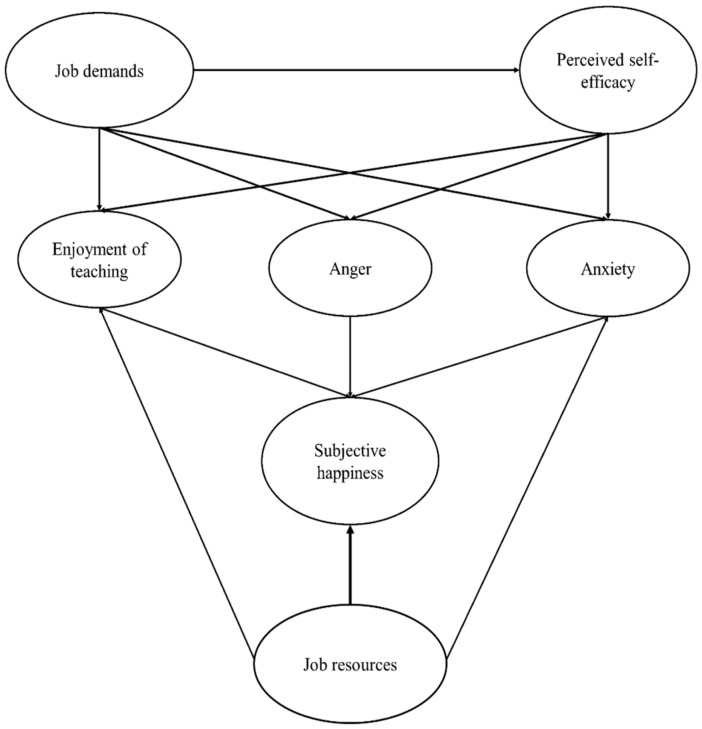
The conceptual model of demands, workplace resources and teaching emotions in relation to teachers’ well-being.

Integrating the job resources–demands model of well-being and the three teaching emotions, namely the enjoyment of teaching, anger, and anxiety, we propose a theoretical model of teachers’ well-being, integrating subjective happiness as an endogenous variable (see Figure 1). The model presumes that the perceived level of job demands (independent variable) influences the perception on self-efficacy (mediator variable), and both variables influence the enjoyment of teaching, anger and teaching anxiety (endogenous variables). Moreover, the teachers’ emotions mediate the job demands and self-efficacy variables’ influence on subjective happiness. Complementing this, the teachers’ subjective happiness and teaching emotions shall be influenced by another exogenous, independent variable, the resources provided by the professional environment.

The research study we propose was guided by the following research hypotheses:

- H1: The perceived level of job demands negatively influences the perception of self-efficacy and both variables influence the enjoyment of teaching, anger and teaching anxiety;
  ◦ H1a: Job demands will have a positive influence on anger and anxiety;
  ◦ H1b: Job demands will have a negative effect on enjoyment of teaching;
- H2: Personal resources as well as job resources have a significant and positive effect on the teachers’ subjective happiness;
  ◦ H2a: Perceived self-efficacy will be positively related to enjoyment of teaching and to teachers’ subjective happiness and negatively associated with anger and anxiety;
  ◦ H2b: Job resources will be positively related to enjoyment of teaching and to teachers’ subjective happiness and negatively associated with anger and anxiety;
  ◦ H2c: Enjoyment of teaching positively influences the subjective happiness of teachers.

## 4. Materials and Methods 

### 4.1. Participants and Sampling Design

The current research study followed an empirical cross-sectional research design. Non-probability convenience-based sampling design was applied, aiming at maximizing the number of responses. The reference population considered for selecting the sample consisted of pre-university Romanian teachers. In order to select the sample, formal letters were sent to all the school inspectorates in the country and in the capital city (regional decentralised bodies responsible for the supervision and control of the implementation of educational policies and strategies), asking for institutional support in disseminating the invitation to survey. Twenty-five inspectorates responded positively to our call for participation. The sample resulted included 1092 teachers, conducting teaching activities at the following educational levels: pre-school (7.5%), primary school (25.4%), lower secondary (47.6%), upper secondary (40.8%) and postsecondary nontertiary (3.8%). In terms of gender distribution, 87.7% of the respondents in the sample were female, reflecting the over-representation of female teachers nationwide. Most of the participants (61.5%) were aged 31–50 years ($M_{age}=39.7;\;\sigma =10.01$). The Romanian educational system classifies the teaching career according to four levels: debutant teacher, definitive teacher, 2^nd^ degree teacher, and 1^st^ degree teacher. A total of 65.3% of the respondents were 1^st^ degree teachers, while 6% were debutants. Similar percentages (14.6% and 14.2%, respectively) are recorded for definitive teachers and 2^nd^ degree teachers. A total of 82.14% of the respondents lived in an urban area and 17.86% in the rural area. The geographical distribution of the sample is unbalanced, with a clear over-representation of respondents who were residents in the capital region (54.7%). Nevertheless, respondents from 25 counties out of 42 were sampled.

### 4.2. Instruments 

In order to encompass all the dimensions underpinning the theoretical model, the authors have designed an omnibus questionnaire following a three-stage process: (1) literature review and operationalisation of the theoretical dimensions; (2) qualitative and quantitative pretesting; (3) development of the final version. The first version of the questionnaire was qualitatively and quantitatively tested during the second stage. Two focus groups with 15 participants were organized. The research instrument was analysed based on the following criteria: item clarity, difficulty, and assessment scale. During group discussions, the participants have suggested reviewing some of the items related to teaching emotions. The improved version was quantitatively tested by 42 teachers with the same characteristics as the final sample. Cronbach–alpha coefficients, inter-item correlations, and exploratory factor analysis have been applied to test the reliability and internal validity of the questionnaire.

In the following, the variables included in the study and the approach designed to measure them are described. 

#### 4.2.1. Job Demands 

Similar to the approach proposed by Bermejo-Toro, Prieto-Ursua and Hernandez [33], the questionnaire included items presenting three types of situations that the scientific literature describes as stressful for teachers: (1) disruptive behaviours of the students (e.g., disobedience, absenteeism); (2) overwork and insufficient time for carrying out the work tasks (e.g., a high number of work tasks and the perception that their completion is impossible, such as preparing the lessons, correcting the tests, and others); (3) conflictual situations (e.g., in relation to the parents or school leadership). 

The authors have operationalised the three situations in five items: two items for the first and third situation, respectively, and one item for the second situation (see Table 1). To evaluate the demand level, each of the designed situations was accompanied by the question: “To what extent do you believe that this situation is a tensed one for you?” The answer to the question was recorded on a 5-point Likert scale, varying from 1 = to a very little extent, 2 = to a little extent, 3 = to a moderate extent, 4 = to a large extent, 5 = to a very large extent. 

#### 4.2.2. Job Resources 

The job resources were defined through the following variables (Table 2): safety and financial comfort; colleagues’ respect; social prestige; autonomy in making decisions; contexts for expressing professional and transversal competences; anxious contexts; feedback; social support (from co-workers and from the leadership team); job variety (task and skill variety); students’ respect. Each of the abovementioned variables was measured on a 5-point Likert scale (1 = to a very little extent, 2 = to a little extent, 3= to a moderate extent, 4 = to a large extent, 5 = to a very large extent). The items in the scale have been designed by the authors.

#### 4.2.3. Perceived Self-efficacy 

In order to measure the self-efficacy perception, the authors have formulated contextual items to present the potentially anxiogenic situations, building upon the approach proposed by Bermejo-Toro et al. [33]. Each item was associated with an item, formulating in a projective manner the capability to provide an adequate answer to the situation described. Each of the five items presenting potentially stressful situations was followed by the question: “When you find yourself in a situation similar to the one above, to what extent do you believe you can manage it?” [33]. The answer to the question was recorded on a 5-point Likert scale (1 = to a very little extent, 2 = to a little extent, 3= to a moderate extent, 4 = to a large extent, 5 = to a very large extent). 

#### 4.2.4. Subjective Happiness of the Subjects 

This variable was measured through nine items, representing the short version of the Oxford Happiness Questionnaire (OHQ) [88] (see Table 3). Each of the nine items was evaluated on a 5-point Likert scale, where 1 = to a very little extent, 2 = to a little extent, 3= to a moderate extent, 4 = to a large extent, 5 = to a very large extent. The questionnaire has been previously validated on Romanian subjects (see the work of Hendres [89] and Balgiu [90]).

#### 4.2.5. Teaching Emotions 

The teaching emotions scale was adapted starting from the questionnaire authored by Frenzel et al. [85], Teachers’ Emotions Scale (TES). After qualitative and quantitative pre-testing, eleven items were kept (not twenty-four, as in the original version), some of them being reformulated (1 = to a very little extent, 2 = to a little extent, 3= to a moderate extent, 4 = to a large extent, 5 = to a very large extent), see Table 4.

The initial version of TES includes items addressing three emotions (enjoyment of teaching, anxiety, and anger), organised in two categories: general contexts and specific contexts, defined by referring the subject filling in the questionnaire to a certain group of students. The answers were recorded on a 5-point Likert (scale: 1 = to a very little extent, 2 = to a little extent, 3= to a moderate extent, 4 = to a large extent, 5 = to a very large extent). 

### 4.3. Procedure 

The resulting questionnaire was self-administered online through the SurveyGizmo® platform. In order to reach pre-university teachers throughout the country, formal letters were sent to all 42 school inspectorates in the country. They have been invited to disseminate the invitation to study to all the schools they coordinate. The invitation included a link where interested teachers could fill out their e-mail address in order to receive the questionnaire. The resulted e-mail list was checked and validated by using the online email-checker.net platform. The end of this process resulted in an e-mail database with approximately 3200 entries. In the next stage, the authors prepared and launched the e-mail invitation to survey through the MailChimp® platform. The completion rate was about 35%. The participation was voluntary and anonymous. No personal information that could lead to the identification of subjects was collected, and the e-mail addresses weren’t associated with the collected data in any way. Subjects who did not consent to participate in the research had the choice not to fill out the survey. A statement of the nature, purposes, and expected duration of the research was included in the beginning of the survey and in the cover e-mail containing the link to the questionnaire. In addition, the names and the affiliation of the researchers were mentioned. To assure that participation was voluntary, a statement of implied consent was included in the questionnaire: ‘Your willingness to complete the questionnaire indicates your consent to participate in this study’. Teachers received a reassurance of use of their responses solely for research purposes. The Ethics Committee at the institutional level approved the research through the letter of approval no. 21769/25/10/2018.

The data were analysed by using the SPSS v25.0 and Amos software (IBM, New York, NY, USA), and the analysis implied descriptive and inferential statistics, and structural equation modelling. 

Principal Axis Factoring was applied in an exploratory manner to deduce the main factors described by the variables in the study and to reduce the data to a reasonable number of variables. A factor analysis was performed for each dimension included in the theoretical model. Cronbach’s alpha coefficient ranged from 0.725 to 0.865 (see Table 5), indicating a good to very good internal consistency of each dimension included in the model. 

By applying the factor analysis, we reduced the number of variables and tested the unidimensionality of each latent variable, but the analyses were separated, and the method was exploratory. Thus, structural equation modelling was applied to validate the results of the factor analysis and to test the hypotheses. Following the recommendations of Hooper et al. [91], the normed/relative chi-square ($X^{2}/df)$ could take values between 2 and 5. Other fit indices were also computed and analysed: RMSEA, GFI, AGFI, RFI, and TLI. MacCallum et al. [92] and [65] suggest that an RMSEA value between 0.05 and 0.08 can be considered a fair fit. More recent scholar opinions report values less than 0.07 [93] in order to consider a correct fit of the model. Regarding the GFI, AGFI, CFI and TLI indices, the values should be close to the 0.95 threshold [91]. Values between 0.85 and 0.95 indicate a satisfactory fit of the model to empirical data [92]. In this paper, we followed the criteria and acceptable thresholds suggested by Coughlan et al. [91] and MacCallum et al. [92].

## 5. Results

The present paper aimed at developing a comprehensive model of pre-university teachers’ well-being. The proposed model includes a relevant and significant selection of variables: job demands; job resources, perceived self-efficacy, subjective happiness, and teaching emotions. Hence, the model postulates that the perceived level of job demands (independent, exogenous variable) affects the perceived self-efficacy (mediator variable) and both variables influence the teaching emotions (dependent variables). Complementing this, the model proposes another exogenous variable—job resources—influencing teachers’ happiness and enjoyment of teaching. In addition, the model draws a relationship between enjoyment of teaching and subjective happiness. 

Table 6 presents descriptive statistics for the observed variables included in the statistical model.

Mean analysis suggested that respect (RLM2), social and professional support (RLM8), task variety (RLM9), and feedback provided by fellows (RLM7) are the most important resources of schools as professional environments. On a contrary, the respondents reported a lack of financial safety and comfort (RLM1). The findings pointed out that teaching is a source of positive emotions. The variables associated with the enjoyment of teaching (JOY_1, JOY_2, JOY_3, JOY_4) have mean scores varying from 4.37 to 4.75. Kruskal–Wallis H Test was conducted to examine the differences in teaching enthusiasm (JOY_1) according to teachers’ career level. No significant differences were found: $X^{2}\left(3\right)=1.69,\;p=0.63$, with a mean rank enthusiasm score of 559.35 for debutant teachers and 549.34 for first degree teachers. Lower mean scores were computed for negative emotions associated with teaching, namely anxiety (ANX_1, ANX_2, ANX_3) and anger (ANG_1, ANG_2, ANG_3, ANG_4). Relative frequency analysis indicated that 35% of the subjects feel worried during teaching activities to a great extent. There are no significant differences related to the teaching experience variable, as Kruskal-Wallis H test showed ($X^{2}\left(3\right)=3.42,\;p=0.33$, with a mean rank score of 572.18 for debutant teachers and 535.66 for more experienced subjects (first degree teachers).

In regard to respondents’ subjective happiness, the mean score computed for the whole sample is M=3.89, st.dev.=0.57, Minimum=1.89, Maximum=5). These values suggest an optimal level of individual subjective happiness. Statistically significant differences were found between female and male subjects: U=49406, p=0.00), indicating that female teachers reported higher levels of happiness. 

The statistical model proposed in this paper postulates a relationship between job resources and demands. To research job demands, typical stressors were operationalised: student disruptive behaviour, overwork and lack of time for job duties, conflicts with peers and parents. As the mean analysis indicates (Table 6), the main stress generator factors are conflicts (SM_2) and disobedient students (SM_3). Statistically significant differences were found for teachers in rural and urban areas (U=7.67, p=0.006), concluding that student disobedience is more significantly perceived as a stressor by teachers in rural schools. In terms of perceived self-efficacy, the Mann-Whitney U test showed no statistically significant differences between urban and rural residents (U=8.67, p=0.85).

To reduce the number of variables included in the statistical model, principal axis factoring with Varimax rotation was conducted. The Kaiser–Meyer–Olkin (KMO) test confirmed that the data were suited for factor analysis: KMO=0.889. In addition, the Bartlett’s test of sphericity was applied to test the adequacy of the data to factor analysis. The test was found to be statistically significant, p<0.01. Seven factors with eigenvalues greater than 1 [94] were identified and retained in the model (Table 7). Most of the extracted communalities ($h^{2}$) were above 0.4. Although there were communalities lower than the 0.4 threshold, we have decided to retain them in the analysis due to theoretical relevance.

Using the regression method, seven factor scores have been extracted. The factor scores were used to compare different subgroups according to residence, gender, teaching experience. Table 8 presents the correlations between the factor scores computed during the factor analysis. All the correlations are statistically significant at the 0.01 level.

As anticipated in research hypothesis H1a, job demands are positively and significantly correlated with anger and anxiety. Antithetically, the H1b research hypothesis stated a negative relationship between job demands and enjoyment of teaching. The correlation between the two variables is negative and statistically significant, as data in Table 8 suggest.

Statistically significant differences were found for the job resources variable according to rural–urban residence: $t\left(1090\right)=2.716,\;p=0.007$, pointing out that teacher in rural areas perceive that schools offer fewer resources to support their work. In addition, One-Way ANOVA with Bonferroni correction was applied to test differences between subgroups according to their level of career. The F test is statistically significant: $F\left(3,1089\right)=3.085,p=0.02,$ concluding that new teachers perceived school professional environments as more resourceful than experienced teachers (p=0.03, the mean difference is significant at the 0.05 level). There were no significant differences for gender ($t\left(1090\right)=2.647,\;p=0.1)$ and for educational level.

In regard to job demands, differences were found between subjects teaching in rural and urban areas: $t\left(1090\right)=2.228,\;p=0.02,$ suggesting that teachers in rural schools perceived a higher burden of job demands than those teaching in urban areas. Despite new teachers perceiving schools as more resourceful environments than experienced teachers did, no significant differences were computed for job demands, teaching emotions, and subjective happiness variables. As expected, first degree teachers felt more self-efficacious than their younger colleagues, as the *One-Way ANOVA* analysis with Bonferroni correction confirmed: $F\left(3,1089\right)=2.884,p=0.03.$

In order to confirm the findings of the exploratory factor analysis, a two-step structural equation modelling with maximum likelihood estimation was performed. The initial statistical model tested with Amos (IBM, New York, NY, United States), included 40 observed variables, five unobserved endogenous variables (namely subjective happiness, enjoyment of teaching, anger, anxiety, and perceived self-efficacy), and two unobserved exogenous variables (job demands and job resources). The initial structural model displayed acceptable fit (Table 9): $X^{2}\left(df=728,\;N=1092\right)=3132.57,\;p<0.05$). The normed chi-square is 4.303, being close to the upper threshold of 5. The RMSEA value can be considered very good. The RFI index is lower than 0.8 (RFI=0.79), but the other indices are above the lower threshold. 

Mardia’s coefficient is 334.6 and the critical ratio (c.r.) = 95.3, indicating a significant non-normality. Thus, the data were bootstrapped with 1000 draws at 95% bias-corrected confidence level. The standardized values computed after bootstrapping (estimates, *p* values, standard errors) are reported in Table 10. For the unstandardized estimates and standard error, please see Appendix A.

The standardised indirect (mediated), statistically significant (p<0.001) effect of job resources on subjective happiness is 0.217. This is in addition to the direct (unmediated), statistically significant (p<0.001)) effect of 0.296 that job resources have on subjective happiness. The standardised indirect (mediated) effect of job demands on subjective happiness is −0.153. The variable job demands also has an indirect, negative and standardised effect on enjoyment of teaching of −0.160. Perceived self-efficacy has also a positive indirect effect on subjective happiness of 0.066 (p<0.001). In addition to this is a direct standardised effect on the enjoyment of teaching variable (0.176, p<0.001).

Re-specifying the initial structural model implied a number of changes in the model. Thus, some variables and the relationships between them have been eliminated from the initial model. We have decided to keep the enjoyment of teaching (as mediator variable) and to eliminate negative emotions from the model (teaching anxiety and anger). The two categories of emotions refer to two modalities of influencing subjective happiness: one negative modality and one positive modality. The negative modality, represented by anxiety and anger emotions, proved to be less significant within the model: due to this we decided to keep in the model the positive emotion called enjoyment of teaching. Although some of the observed variables associated with subjective happiness (OHQ_1_rev, OHQ_4_rev, OHQ_7, OHQ_8, and OHQ_9_rev) had low communalities, we have decided to keep them in the model due to theoretical relevance. Table 11 synthetizes the fit indices of the re-specified model. By eliminating the negative teaching emotions from the model, most of the fit indices increased: $X^{2}\left(df=489,\;N=1092\right)=2132.92;RMSEA=0.05;GFI=0.90;AGFI=88;RFI=0.85;TLI=0.87;CFI=0.89$. Therefore, the model can be considered adequate. 

Standardized path coefficients, standard errors, and bootstrapping results for the re-specified model are presented in Table 12 (please see Appendix A (Table A1 and Table A2) for the unstandardized values).

**Figure 2:**
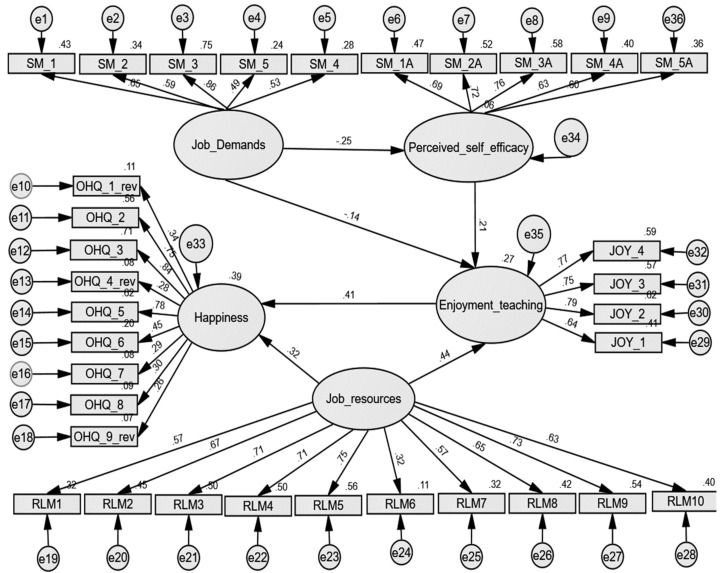
Structural equations re-specified model of teacher well-being (standardized estimates).

Figure 2 presents the path diagram of the re-specified model, graphically replicating the postulated relations between variables. The diagram confirms the values calculated through the model adequacy indices. All the path coefficients are significant (p<0.001).

The noteworthy feature of this model is the positive relationship between the enjoyment of teaching and subjective happiness variables (H2c hypothesis), and between job resources and enjoyment of teaching (H2b hypothesis), as illustrated by the unstandardized path coefficients of 0.23 and 0.33, respectively. The standardised coefficients indicate a stronger relationship between enjoyment of teaching and subjective happiness (0.41), confirming research hypothesis H2c, and job resources and enjoyment of teaching (0.44), validating the assumption stated in the hypothesis H2c. Moreover, there is a negative relationship between job demands and perceived self-efficacy (unstandardized path coeffcient=−0.17, standardized coefficient=−0.25). The measurement portion of the model is good: $0.27<R^{2}<0.39),$ indicating that enjoyment of teaching and job resources variables account for 39% of the variance in subjective happiness. The two variables are significant predictors of the endogenous variable subjective happiness (p<0.001). Job demands and perceived self-efficacy variables explain 27% of the variance in the enjoyment of teaching, being significant predictors of the enjoyment of teaching (p<0.001). The lowest $R^{2}=0.06$ value in the model indicates that job demands explain 6% of the variance of the perceived self-efficacy variable. 

Within the re-specified model, job resources have a standardized, positive, and significant mediation effect on subjective happiness of 0.182. Moreover, job resources have a significant influence on both enjoyment of teaching and on teachers’ subjective happiness: the relations are positive and statistically significant (p<0.001). The research revealed that, in the case of Romanian teachers, job resources have a more powerful influence on the enjoyment of teaching and subjective happiness (standardized direct effect is 0.439) than self-efficacy has (standardized direct and negative effect of −0.137). The direct effect of job resources on happiness is 0.320 and on enjoyment of teaching is 0.439. In addition, job resources have an indirect effect of 0.12 on subjective happiness, resulting in a total effect of 0.50. Therefore, the computed scores endorse the decision to accept the research hypothesis H2: job resources have a significant effect on the teaching staff well-being, in terms of subjective happiness: $\left(0.44*0.41\right)+0.32=0.50.$ It is to be noted that job resources also have a moderate indirect effect on the OHQ_3 (‘I am well satisfied about everything in my life’) observed variable of 0.423.

The saturation coefficients indicate that creating challenging contexts to address various professional competences (RLM5) is the most influential vector of the job resources dimension (0.75), followed by task variety—RLM9 (0.73), and autonomy—RLM4 (0.71). Nevertheless, the job resources significantly influence the enjoyment of teaching, whose most influential vector is enthusiasm (0.79). 

One central element of the model is the relation between the enjoyment of teaching and the subjective happiness of subjects. The relation between the two variables is positive, statistically significant, and moderate in intensity (standardized coefficient path=0.41). The total effect of the enjoyment of teaching positive emotion on subjects’ happiness is 0.415. This value confirms the H2c hypothesis: the enjoyment of teaching influences the subjective happiness of the teachers.

Job demands have a direct, standardized effect of $\left(-0.25\right)$ on the perceived self-efficacy and an indirect effect on the enjoyment of teaching of $\left(-0.053\right)$, the relation being mediated by the perceived self-efficacy. The association of job demands and perceived self-efficacy is negative (standardized coefficient=−0.25)  and significant p<0.001),  as stated in research hypothesis H1. Therefore, by increasing the job demands the perceived self-efficacy would be decreased. A similar result is also found in other, similar researches [33,87]. The computed score suggests that the perceived self-efficacy (seen in this context as a personal resource) has a lower influence on subjective happiness (indirect effect of 0.088) and on the enjoyment of teaching (direct effect is 0.21) compared to the resources provided by the professional environment. In line with the previously presented findings, disobedient students (SM_3) represent the most impactful and stress generator variable (0.86) in terms of job demands. 

## 6. Discussion

The aim of this study was to extend the dynamic equilibrium well-being model and to design a teacher well-being model that values the role of personal resources in the prediction of subjective happiness. In order to do so, self-efficacy and teaching emotions were included in the model as personal resources. Generally, the research results have confirmed research hypotheses 1 and 2, since job demands, job and personal resources are significant predictors of teacher subjective happiness. The research has drawn a line of influence starting with the perceived level of job demands, whose effect on subjective happiness is mediated by the teachers’ self-efficacy. Influenced by job resources, the enjoyment of teaching plays a part in teacher subjective happiness, also influenced by the job resources that teachers benefit from.

To outline, structural equation modelling applied to both initial and re-specified models provided evidence that empirical data support the conceptual model initially designed. More precisely, the results showed that both personal and job resources have a positive and significant effect on subjective happiness, seen as an indicator of teacher well-being (hypothesis 2). The paths computed between personal and job resources and teacher subjective happiness have moderate values. In line with prior findings, the current research has suggested that task variety, autonomy, and the value of the profession are predictors of teacher subjective happiness [33,59,63,95]. 

One particular feature of the model is that it takes into consideration the importance of teaching positive emotions (hypothesis H2c). Therefore, it is important to discuss the role of positive teaching emotions on teacher subjective happiness. As the research has revealed, the direct effect of the enjoyment of teaching on subjective happiness is greater than the effect job resources have on the same variable. As Fredrickson suggests [79,80], the effect of positive emotions can be explained by the broaden and build theory, stating that the higher the positive emotions individuals attribute to themselves, the higher the chance to build positive aspects of the self [80]. Along the same lines, Buonomo et al. ([96] emphasise the role of positive emotions in predicting teacher self-efficacy. According to our H2a hypothesis, findings have shown that enjoyment of teaching is positively related to self-efficacy. As other studies have reported, positive emotional states are likely to be associated to subjects perceiving themselves as more efficacious [96,97,98]. In addition, the work of Fredrickson and other subsequent studies have strongly supported the hypothesis that positive emotions can boost other personal resources [77,79,81]. 

Moreover, the current research is in agreement with recent studies putting forward the role of job resources in increasing intrinsic motivation and proactive behaviour at work [59,63]. In our study, job variety, teacher autonomy, and the value of the profession seem to play a more important role, confirming hypothesis H2b. These results led us to consider a design of a school professional environment where job resources (more specifically, autonomy and job variety) are used to self-generate authentic, contextually relevant positive emotions, eventually boosting teacher subjective happiness. In support of this, Wang et al. [44] advocate the need to increase teacher autonomy. As OECD analyses [43] suggest, highly performant countries and economies foster high levels of teacher autonomy. Autonomous work in the classroom is also seen as the core of the teaching profession [99]. Therefore, an ideal work environment would offer not only financial comfort, but would create a culture of sharing and respect, where teachers could benefit from task variety, feedback, and social support. This finding is in line with other scholar’s opinions suggesting that a culture of sharing and learning could improve teacher professionalism and learning outcomes alike [45,54]. Garcia and Weiss [54] point out that novice and veteran teachers largely don’t get access to proper resources to prepare their teaching practice. Developing communities of practice and supporting professional networks membership could be beneficial to enriching the professional environment. In addition, a valuable solution to boosting the school environment’s resources would consist of mentoring activities and professional coaching, due to the significant relevance of feedback, seen as an impactful resource. Additionally, the study has found that novice teachers perceive schools as more resourceful organisations than their experienced colleagues do. A particular characteristic of the teachers who expressed these opinions is that they reported higher levels of enthusiasm (a dimension of enjoyment). Thus, induction and mentorship programmes addressing novice teachers could exploit this personal resource, as Darlin–-Hammond have suggested [52]. 

Their variety would be beneficial for teacher well-being. The research of Bermejo-Toro et al. [33] identified a higher contribution of self-efficacy compared against the one of job resources. 

We believe that the current findings are an important contribution to explaining the role of challenges and resources on teacher subjective happiness, here understood as a dimension of well-being. As previously stated, well-being is a multidimensional construct and subjective happiness is one of its facets. However, the study stresses the need for more research on teaching positive emotions and their effect on psychological well-being.

## 7. Practical Implications and Contributions of the Study

The present research sought to test two research hypotheses, as follows:

- H1: The perceived level of job demands negatively influences the perception on self-efficacy and both variables influence the enjoyment of teaching, anger and teaching anxiety;
  ◦ H1a: Job demands will have a positive influence on anger and anxiety;
  ◦ H1b: Job demands will have a negative effect on enjoyment of teaching;
- H2: Personal resources as well as job resources have a significant and positive effect on the teachers’ subjective happiness;
  ◦ H2a: Perceived self-efficacy will be positively related to enjoyment of teaching and to teachers’ subjective happiness and negatively associated with anger and anxiety;
  ◦ H2b: Job resources will be positively related to enjoyment of teaching and to teachers’ subjective happiness and negatively associated with anger and anxiety.

Thus, personal and job resources proved to play a pivotal role in achieving subjective happiness A significant contribution of the study relies on personal resources (perceived self-efficacy and teaching emotions) to design a teacher well-being model. A similar study was conducted by Bermejo-Toro, Prieto-Ursua and Hernandez [33], but the tested model does not include teaching emotions.

The results of the study could have an influential impact at individual and institutional levels alike, leading to significant improvements in well-being. The emotions of teachers are considered relevant not only for their own well-being but also for the functioning of classrooms [85] and, as Chen [75] pointed out, the research on how emotions impact teaching approaches is rather limited A notable contribution of the study is that is emphasizes the role of the enjoyment of teaching in achieving the outcome of happiness. Thus, job resources could boost the enjoyment of teaching, which, in turn, forecasts the subjective happiness of teachers. Moreover, the model points out the nature of the job resources that could be considered to design teacher education and insertion programmes [28]. Providing feedback and encouraging teacher autonomy could be critical components throughout the process of teaching career development [45,54]. Moreover, further research could focus on urban–rural and regional differences in relation to the teachers’ representations on the job resources they benefit from.

In regard to job demands, overwork proved not to be the most impactful stressor, as the study emphasized. Managing student disruptive behaviour is a source of distress. This issue could be better tackled through better initial and continuous training. 

Summarizing the findings of the study, a noteworthy contribution is that it shows that, in order to improve well-being at work, it is necessary to boost and rethink the job resources, both in terms of quantity and quality.

## 8. Limitations of the Study

First, it is important to mention that sample and sampling limitations need to be considered in the interpretation of the results. Generalisation of the results is not possible due to the sampling procedure. Moreover, the sample is geographically unbalanced, and this could result in relevant differences. In addition, the completion rate is 35%. Although the present study did not identify significant differences between teachers at different educational levels, further research could explore such a research question.

Secondly, although the cross-sectional design applied in the current study allows inferences about the relationship between variables, it does not allow the identification of cause–effect relationships. A longitudinal approach could better address the mediation effects, as mediations on cross-sectional data are known to be biased because of autoregressive effects [100]. Nevertheless, the use of structural equation modelling allowed the study of complex relations between the variables in the model, resulting in a comprehensive understanding of teacher well-being. 

Thirdly, we acknowledge that some limitations may arise as consequences of the administered scales and data collection procedures. Since the questionnaire was administered through the WAPI method, we were interested in some additional metrics of the research instrument, namely the length and the fatigue score. This led to preferring the short version of the Oxford Happiness Questionnaire. Despite these additional measures, the response rate was 35%. Thus, the low response rate could be responsible for potential bias of the collected data. Additionally, a distinction between the effects associated to trait- and state emotions could be beneficial, as they have different impact on teachers’ experiences, as Goetz et al. have pointed out [101]. 

Finally, it is important to note that even though this model is in agreement with the empirical data and provides a theoretically consistent set of findings, there may be other equivalent models that fit the data equally well.

## 9. Conclusions

This research aimed to design and test on empirical data a model of teachers’ well-being, integrating the personal resources represented by the enjoyment of teaching and perceived self-efficacy within the framework of the dynamic equilibrium model refined by Dodge et al. [16] . The results of the study show that, in order to improve teacher well-being, it is important to consider both job and personal resources such as self-efficacy and positive teaching emotions. The enjoyment of teaching, as a positive emotion, is positively influenced by job resources and, in turn, impacts subjective well-being, preventing distress and exhaustion. Although the research revealed that autonomy and job variety may be particularly important, it should be noted that all job resources in the model are correlated, meaning that feedback, value of the profession and relationships could also be beneficial to teacher well-being. As the research proved, there is a clear need to focus on job resources rather than job demands and to empower teachers through effective feedback and supportive relationships. A particular contribution of the study is that it emphasises the role of positive emotions in the dynamic development of subjective happiness. Thus, the enjoyment of teaching could forecast valuable outcomes such as subjective happiness of teachers. 

The research validates and creates a reflection environment in regard to the management and self-management of the teaching career. The professional construction is an approach of remarkable complexity, with major implications at both systemic and personal levels. The study on teachers’ emotions and well-being might influence the approach to the processes involved by initial and continuous professional training, and teaching staff employability and professionalism, thus granting sustainability to this approach, according to the provisions of the Declaration of Brussels: “We launch an appeal towards providing initial and continuous training for teachers from public funds, transparent recruitment and selection, decent work condition, professional autonomy and an attractive career path for teachers, educators, trainers, and school directors” [102]

## Figures and Tables

**Table 1:** Teacher job demands scale.

Code	Items
SM_1	One of the students in your class has his headphones on and refuses to solve the proposed learning tasks. To what extent is this a tense situation for you?
SM_2	A parent comes to school and says she is deeply unhappy with your teaching activity. She worries that her son will not pass the national exams. To what extent is this a tense situation for you?
SM_3	You are in the middle of a teaching activity. Two of the students in the classroom chat with each other, looking constantly at their cell phones. Initially, you ignore their behaviour. Later, you attract their attention, but they keep talking. To what extent is this a tense situation for you?
SM_4	It is the final examination period. You have to grade tests and final assignments. The school principal informs you the inspector will pay a visit to your school and you must organise a demo lesson. The visit is scheduled for the day after tomorrow. To what extent is this a tense situation for you?
SM_5	The school principal assigned you and one of your colleagues an administrative task. The colleague informs you that he cannot handle the task. You are in the position to manage it by yourself. To what extent is this a tense situation for you?

**Table 2:** Teacher job resources scale.

Code	To What Extent the Teaching Profession Offers You the Following:
RLM1	Safety and financial comfort
RLM2	Colleagues’ respect
RLM3	Social prestige
RLM4	Autonomy in making decisions
RLM5	Variety of skills.
RLM6	Anxious contexts
RLM7	Feedback from peers and supervisors
RLM8	Support from colleagues and supervisors
RLM9	Variety of job tasks
RLM10	Students’ respect

**Table 3:** Teacher subjective happiness scale [88].

Code	Below Are Several Statements about Happiness. To What Extent Would You Agree with Each of the Following?
OHQ_1_rev	I don’t feel particularly pleased with the way I am. (R)
OHQ_2	I feel that life is very rewarding
OHQ_3	I am well satisfied about everything in my life
OHQ_4_rev	I don’t think I look attractive (R)
OHQ_5	I am very happy
OHQ_6	I find beauty in some things
OHQ_7	I can fit in everything I want to
OHQ_8	I feel fully mentally alert
OHQ_9_rev	I do not have particularly happy memories of the past (R)
Note: (R) items were reversed scored.	

**Table 4:** Items of the teaching emotions scale.

Category	Code	Adapted Items
Enjoyment of teaching	JOY_1	Generally, I enjoy teaching.
	JOY_2	Generally, I feel good when I teach, so I prepare my lessons with enthusiasm.
	JOY_3	Many times, I have reasons to feel joyful while I teach.
	JOY_4	Generally, I teach with enthusiasm.
Anger	ANG_1	I often have reasons to feel angry while I teach.
	ANG_2	I often feel irritated by my students’ behaviour while I teach.
	ANG_3	I often get angry while I teach.
	ANG_4	I frequently wish to have chosen a different profession at the end of a lesson.
Anxiety	ANX_1	Generally, I do not feel comfortable while teaching.
	ANX_2	I often feel worry that the teaching activity does not go so well.
	ANX_3	When I prepare my lessons, I start to worry for the way thigs would go.

**Table 5:** Values of Cronbach’s alpha coefficients.

Dimension	Cronbach’s Alpha
Job demands	α = 0.761, N items = 5
Perceived self-efficacy	α = 0.811, N items = 5
Enjoyment of teaching	α = 0.717, N items = 4
Anxiety	α = 0.882, N items = 3
Anger	α = 0.805, N items = 4
Subjective happiness	α = 0.725, N items = 9
Job resources	α = 0.865, N items = 10

Source: Developed by the authors based on the collected data.

**Table 6:** Descriptive statistics of the variables included in the model.

Variables	Minimum	Maximum	Mean	Std. Deviation	Skewness	Kurtosis
RLM1	1	5	2.53	1.035	0.207	−0.485
RLM2	1	5	3.84	0.970	−0.727	0.168
RLM3	1	5	3.05	1.154	−0.158	−0.757
RLM4	1	5	3.17	1.039	−0.262	−0.470
RLM5	1	5	3.61	0.992	−0.445	−0.159
RLM6	1	5	2.99	1.165	0.051	−0.784
RLM7	1	5	3.50	0.922	−0.387	0.000
RLM8	1	5	3.75	1.034	−0.732	0.117
RLM9	1	5	3.62	1.051	−0.504	−0.287
RLM10	1	5	3.61	1.092	−0.620	−0.221
JOY_1	1	5	4.75	0.543	−2.759	10.443
JOY_2	1	5	4.55	0.666	−1.693	3.908
JOY_3	1	5	4.37	0.770	−1.271	1.811
JOY_4	1	5	4.53	0.678	−1.626	3.698
ANX_1	1	5	2.77	1.330	0.094	−1.211
ANX_2	1	5	2.40	1.248	0.462	−0.934
ANX_3	1	5	1.47	1.028	2.443	5.082
ANG_1	1	5	1.66	1.015	1.587	1.820
ANG_2	1	5	2.09	1.085	0.849	0.037
ANG_3	1	5	1.56	0.910	1.841	3.128
ANG_4	1	5	1.49	0.912	2.160	4.418
OHQ_1_rev	1	5	4.25	1.138	−1.392	0.750
OHQ_2	1	5	4.12	0.891	−1.117	1.452
OHQ_3	1	5	3.86	0.897	−0.797	0.646
OHQ_4_rev	1	5	3.99	1.155	−0.861	−0.269
OHQ_5	1	5	3.78	0.912	−0.753	0.599
OHQ_6	1	5	4.36	0.829	−1.549	2.928
OHQ_7	1	5	3.09	1.205	−0.256	−0.813
OHQ_8	1	5	3.54	1.109	−0.720	−0.029
OHQ_9_rev	1	5	4.08	1.091	−1.001	0.086
SM_1	1	5	2.86	1.251	0.100	−0.914
SM_2	1	5	3.09	1.360	−0.147	−1.154
SM_3	1	5	3.07	1.194	−0.183	−0.872
SM_4	1	5	2.95	1.355	−0.034	−1.180
SM_5	1	5	2.77	1.298	0.110	−1.105
SM_1A	1	5	4.30	0.810	−1.230	1.855
SM_2A	1	5	4.21	0.810	−1.156	1.898
SM_3A	1	5	4.24	0.793	−1.039	1.351
SM_4A	1	5	4.30	0.781	−1.145	1.529
SM_5A	1	5	4.27	0.797	−1.069	1.333
Source: Developed by the authors based on the collected data						

**Table 7:** Summary factor analysis and eigenvalues.

Factor	Items	Communalities	Item Loading	%Variance Explained Cumulative	Eigen Value
Job resources	RLM1	0.361	0.559	21.297	8.519
	RLM2	0.487	0.664		
	RLM3	0.534	0.706		
	RLM4	0.523	0.711		
	RLM5	0.552	0.694		
	RLM6	0.146	0.302		
	RLM7	0.319	0.522		
	RLM8	0.43	0.633		
	RLM9	0.538	0.675		
	RLM10	0.426	0.572		
Anger	ANG1	0.554	0.704	29.448	3.260
	ANG2	0.561	0.661		
	ANG3	0.485	0.63		
	ANG4	0.487	0.603		
Perceived self-efficacy	SM1_A	0.452	0.616	35.212	2.306
	SM2_A	0.495	0.679		
	SM3_A	0.566	0.73		
	SM4_A	0.445	0.641		
	SM5_A	0.395	0.598		
Subjective happiness	OHQ_1_rev	0.167	0.281	40.631	2.167
	OHQ_2	0.55	0.651		
	OHQ_3	0.695	0.739		
	OHQ_4_rev	0.152	0.25		
	OHQ_5	0.613	0.7		
	OHQ_6	0.297	0.38		
	OHQ_7	0.159	0.339		
	OHQ_8	0.138	0.294		
	OHQ_9_rev	0.12	0.176		
Enjoyment of teaching	JOY_1	0.43	0.571	45.233	1.841
	JOY_2	0.655	0.73		
	JOY_3	0.572	0.626		
	JOY_4	0.593	0.662		
Job demands	SM_1	0.397	0.599	49.462	1.692
	SM_2	0.345	0.582		
	SM_3	0.712	0.818		
	SM_4	0.384	0.553		
	SM_5	0.325	0.493		
Anxiety	ANX1	0.528	0.691	52.616	1.261
	ANX2	0.636	0.703		
	ANX3	0.204	0.4		
Source: Developed by the authors based on the collected data					

**Table 8:** Correlation matrix of the factor scores computed through factor analysis.

Factor Scores		1	2	3	4	5	6
**1**	Job resources	-					
2	Enjoyment of teaching	0.420 **	-				
3	Job demands	−0.131 **	−0.218 **	-			
4	Perceived self-efficacy	0.233 **	0.281 **	−0.239 **	-		
5	Subjective happiness	0.410 **	0.421 **	−0.156 **	0.227 **	-	
6	Anger	−0.274 **	−0.426 **	0.323 **	−0.273 **	−0.295 **	-
7	Anxiety	−0.048	−0.025	0.153 **	−0.161 **	−0.054	0.053
**. Correlation is significant at the 0.01 level (2-tailed).							

**Table 9:** Goodness of fit indices of the initial teacher well-being model, N = 1092.

	$X^{2}$	DF	$X^{2}/DF$	RMSEA	GFI	AGFI	RFI	TLI	CFI
Initial Model	3132.57	728	4.303	0.05	0.86	0.85	0.79	0.83	0.84
Source: Developed by the authors based on the collected data									

**Table 10:** Initial model’s standardized estimates, standard errors, and bootstrapping results.

			Estimate	P	SE	SE-SE	Bias	SE-Bias	
Perceived_self_efficacy	<---	Job_Demands	−0.276	***	0.044	0.001	0.000	0.001	
Enjoyment_teaching	<---	Perceived_self_efficacy	0.190	***	0.042	0.001	−0.001	0.001	
Enjoyment_teaching	<---	Job_Demands	−0.160	***	0.036	0.001	0.001	0.001	
Anxiety	<---	Job_Demands	0.241	***	0.048	0.001	−0.002	0.002	
Anger	<---	Job_Demands	0.406	***	0.041	0.001	0.000	0.001	
Enjoyment_teaching	<---	Job_resources	0.455	***	0.035	0.001	−0.001	0.001	
Anger	<---	Job_resources	−0.338	***	0.039	0.001	0.000	0.001	
Anxiety	<---	Job_resources	−0.113	***	0.036	0.001	0.000	0.001	
Happiness	<---	Job_resources	0.296	***	0.037	0.001	−0.001	0.001	
Happiness	<---	Enjoyment_teaching	0.347	***	0.043	0.001	0.001	0.001	
Happiness	<---	Anger	−0.145	***	0.047	0.001	−0.001	0.001	
Happiness	<---	Anxiety	−0.086	0.010	0.034	0.001	0.001	0.001	
SM_3	<---	Job_Demands	0.844	***	0.023	0.001	0.000	0.001	
OHQ_9_rev	<---	Happiness	0.260		0.034	0.001	−0.001	0.001	
OHQ_8	<---	Happiness	0.302	***	0.032	0.001	0.002	0.001	
OHQ_7	<---	Happiness	0.284	***	0.034	0.001	0.000	0.001	
OHQ_6	<---	Happiness	0.442	***	0.034	0.001	0.001	0.001	
OHQ_5	<---	Happiness	0.777	***	0.022	0.000	0.000	0.001	
OHQ_4_rev	<---	Happiness	0.280	***	0.034	0.001	0.000	0.001	
OHQ_3	<---	Happiness	0.839	***	0.018	0.000	0.000	0.001	
OHQ_2	<---	Happiness	0.747	***	0.023	0.001	0.000	0.001	
OHQ_1_rev	<---	Happiness	0.340	***	0.032	0.001	−0.001	0.001	
JOY_4	<---	Enjoyment_teaching	0.770		0.028	0.001	0.000	0.001	
JOY_3	<---	Enjoyment_teaching	0.753	***	0.023	0.001	0.000	0.001	
JOY_2	<---	Enjoyment_teaching	0.787	***	0.030	0.001	0.001	0.001	
JOY_1	<---	Enjoyment_teaching	0.639	***	0.035	0.001	0.001	0.001	
RLM10	<---	Job_resources	0.637		0.023	0.001	−0.001	0.001	
RLM9	<---	Job_resources	0.733	***	0.017	0.000	0.000	0.001	
RLM8	<---	Job_resources	0.642	***	0.022	0.000	−0.001	0.001	
RLM7	<---	Job_resources	0.568	***	0.024	0.001	−0.001	0.001	
RLM6	<---	Job_resources	0.329	***	0.035	0.001	−0.001	0.001	
RLM5	<---	Job_resources	0.747	***	0.018	0.000	−0.001	0.001	
RLM4	<---	Job_resources	0.705	***	0.018	0.000	−0.001	0.001	
RLM3	<---	Job_resources	0.707	***	0.019	0.000	−0.002	0.001	
RLM2	<---	Job_resources	0.666	***	0.022	0.000	−0.001	0.001	
RLM1	<---	Job_resources	0.565	***	0.024	0.001	0.000	0.001	
SM_5	<---	Job_Demands	0.512	***	0.037	0.001	−0.002	0.001	
SM_4	<---	Job_Demands	0.545	***	0.034	0.001	−0.001	0.001	
SM_2	<---	Job_Demands	0.573	***	0.028	0.001	−0.002	0.001	
SM_1	<---	Job_Demands	0.649		0.027	0.001	0.000	0.001	
SM_1A	<---	Perceived_self_efficacy	0.687		0.031	0.001	0.001	0.001	
SM_2A	<---	Perceived_self_efficacy	0.720	***	0.029	0.001	0.001	0.001	
SM_5A	<---	Perceived_self_efficacy	0.598	***	0.034	0.001	0.000	0.001	
SM_3A	<---	Perceived_self_efficacy	0.761	***	0.028	0.001	0.001	0.001	
SM_4A	<---	Perceived_self_efficacy	0.630	***	0.038	0.001	−0.002	0.001	
ANX_1	<---	Anxiety	0.640		0.041	0.001	−0.003	0.001	
ANX_2	<---	Anxiety	0.928	***	0.054	0.001	0.007	0.002	
ANX_3	<---	Anxiety	0.258	***	0.037	0.001	0.000	0.001	
ANG_1	<---	Anger	0.688		0.028	0.001	−0.001	0.001	
ANG_2	<---	Anger	0.797	***	0.021	0.000	0.001	0.001	
ANG_3	<---	Anger	0.694	***	0.031	0.001	0.000	0.001	
ANG_4	<---	Anger	0.652	***	0.031	0.001	−0.001	0.001	
*** significant at *p*<0.001								

**Table 11:** Goodness of fit indices of the refined teacher well-being model, N = 1092.

	$X^{2}$	DF	$X^{2}/DF$	RMSEA	GFI	AGFI	RFI	TLI	CFI
Re-specified Model	2132.92	489	4.36	0.05	0.90	0.88	0.85	0.87	0.89
Initial Model	3132.57	728	4.303	0.05	0.86	0.85	0.79	0.83	0.84
Source: Developed by the authors based on the collected data									

**Table 12:** Re-specified model’s standardized estimates, standard errors, and bootstrapping results.

Parameter			Estimate	P	SE	SE-SE	Bias	SE-Bias
Perceived_self_efficacy	<---	Job_Demands	−0.249	***	0.043	0.001	0.000	0.001
Enjoyment_teaching	<---	Perceived_self_efficacy	0.211	***	0.042	0.001	−0.001	0.001
Enjoyment_teaching	<---	Job_Demands	−0.137	***	0.034	0.001	0.001	0.001
Enjoyment_teaching	<---	Job_resources	0.439	***	0.035	0.001	−0.001	0.001
Happiness	<---	Job_resources	0.320	***	0.037	0.001	−0.001	0.001
Happiness	<---	Enjoyment_teaching	0.415	***	0.038	0.001	0.001	0.001
SM_3	<---	Job_Demands	0.863	***	0.023	0.001	0.000	0.001
OHQ_9_rev	<---	Happiness	0.257		0.034	0.001	−0.001	0.001
OHQ_8	<---	Happiness	0.305	***	0.033	0.001	0.002	0.001
OHQ_7	<---	Happiness	0.287	***	0.034	0.001	−0.001	0.001
OHQ_6	<---	Happiness	0.448	***	0.034	0.001	0.001	0.001
OHQ_5	<---	Happiness	0.785	***	0.021	0.000	0.000	0.001
OHQ_4_rev	<---	Happiness	0.277	***	0.034	0.001	0.000	0.001
OHQ_3	<---	Happiness	0.842	***	0.018	0.000	0.000	0.001
OHQ_2	<---	Happiness	0.751	***	0.023	0.001	0.000	0.001
OHQ_1_rev	<---	Happiness	0.338	***	0.033	0.001	−0.001	0.001
JOY_4	<---	Enjoyment_teaching	0.771		0.028	0.001	0.000	0.001
JOY_3	<---	Enjoyment_teaching	0.752	***	0.023	0.001	0.000	0.001
JOY_2	<---	Enjoyment_teaching	0.785	***	0.030	0.001	0.001	0.001
JOY_1	<---	Enjoyment_teaching	0.639	***	0.034	0.001	0.001	0.001
RLM10	<---	Job_resources	0.631		0.023	0.001	−0.001	0.001
RLM9	<---	Job_resources	0.734	***	0.017	0.000	0.000	0.001
RLM6	<---	Job_resources	0.325	***	0.034	0.001	−0.001	0.001
RLM1	<---	Job_resources	0.568	***	0.024	0.001	0.000	0.001
SM_5	<---	Job_Demands	0.491	***	0.038	0.001	−0.001	0.001
SM_4	<---	Job_Demands	0.531	***	0.035	0.001	0.000	0.001
SM_2	<---	Job_Demands	0.585	***	0.027	0.001	−0.002	0.001
SM_1	<---	Job_Demands	0.654		0.025	0.001	0.000	0.001
SM_1A	<---	Perceived_self_efficacy	0.686		0.031	0.001	0.001	0.001
SM_2A	<---	Perceived_self_efficacy	0.720	***	0.029	0.001	0.001	0.001
SM_5A	<---	Perceived_self_efficacy	0.598	***	0.034	0.001	0.000	0.001
SM_3A	<---	Perceived_self_efficacy	0.762	***	0.028	0.001	0.001	0.001
SM_4A	<---	Perceived_self_efficacy	0.630		0.038	0.001	−0.002	0.001
RLM5	<---	Job_resources	0.746		0.018	0.000	−0.001	0.001
RLM4	<---	Job_resources	0.708		0.018	0.000	−0.001	0.001
RLM3	<---	Job_resources	0.709		0.019	0.000	−0.002	0.001
RLM2	<---	Job_resources	0.669		0.022	0.000	0.000	0.001
RLM7	<---	Job_resources	0.568		0.025	0.001	−0.001	0.001
RLM8	<---	Job_resources	0.645		0.022	0.000	−0.001	0.001
Note: *** significant at *p*<0.001								

## Appendix A

**Table A1:** Initial model’s unstandardized estimates and standard errors.

			Estimate	S.E.	C.R.	P
Perceived_self_efficacy	<---	Job_Demands	−0.189	0.027	−6.930	***
Enjoyment_teaching	<---	Perceived_self_efficacy	0.176	0.033	5.258	***
Enjoyment_teaching	<---	Job_Demands	0-.101	0.022	−4.520	***
Anxiety	<---	Job_Demands	0.252	0.048	5.278	***
Anger	<---	Job_Demands	0.346	0.034	10.207	***
Enjoyment_teaching	<---	Job_resources	0.336	0.028	11.886	***
Anger	<---	Job_resources	−0.337	0.036	−9.283	***
Anxiety	<---	Job_resources	−0.139	0.042	−3.297	***
Happiness	<---	Job_resources	0.120	0.021	5.807	***
Happiness	<---	Enjoyment_teaching	0.191	0.031	6.196	***
Happiness	<---	Anger	−0.059	0.017	−3.454	***
Happiness	<---	Anxiety	−0.029	0.011	−2.574	0.010
SM_3	<---	Job_Demands	1.240	0.058	21.224	***
OHQ_9_rev	<---	Happiness	1.000			
OHQ_8	<---	Happiness	1.179	0.184	6.416	***
OHQ_7	<---	Happiness	1.205	0.192	6.272	***
OHQ_6	<---	Happiness	1.287	0.173	7.422	***
OHQ_5	<---	Happiness	2.466	0.299	8.257	***
OHQ_4_rev	<---	Happiness	1.142	0.181	6.322	***
OHQ_3	<---	Happiness	2.609	0.314	8.309	***
OHQ_2	<---	Happiness	2.319	0.282	8.230	***
OHQ_1_rev	<---	Happiness	1.362	0.198	6.882	***
JOY_4	<---	Enjoyment_teaching	1.000			
JOY_3	<---	Enjoyment_teaching	1.111	0.044	24.976	***
JOY_2	<---	Enjoyment_teaching	1.002	0.041	24.669	***
JOY_1	<---	Enjoyment_teaching	0.668	0.033	20.022	***
RLM10	<---	Job_resources	1.000			
RLM9	<---	Job_resources	1.107	0.055	20.098	***
RLM8	<---	Job_resources	0.954	0.054	17.800	***
RLM7	<---	Job_resources	0.753	0.046	16.226	***
RLM6	<---	Job_resources	0.552	0.055	9.993	***
RLM5	<---	Job_resources	1.066	0.052	20.361	***
RLM4	<---	Job_resources	1.054	0.054	19.458	***
RLM3	<---	Job_resources	1.173	0.060	19.688	***
RLM2	<---	Job_resources	0.929	0.050	18.590	***
RLM1	<---	Job_resources	0.841	0.052	16.237	***
SM_5	<---	Job_Demands	0.818	0.061	13.430	***
SM_4	<---	Job_Demands	0.909	0.064	14.162	***
SM_2	<---	Job_Demands	0.960	0.061	15.672	***
SM_1	<---	Job_Demands	1.000			
SM_1A	<---	Perceived_self_efficacy	1.000			
SM_2A	<---	Perceived_self_efficacy	1.050	0.052	20.017	***
SM_5A	<---	Perceived_self_efficacy	0.857	0.053	16.200	***
SM_3A	<---	Perceived_self_efficacy	1.086	0.052	20.899	***
SM_4A	<---	Perceived_self_efficacy	0.885	0.052	16.894	***
ANX_1	<---	Anxiety	1.000			
ANX_2	<---	Anxiety	1.359	0.140	9.700	***
ANX_3	<---	Anxiety	0.311	0.040	7.858	***
ANG_1	<---	Anger	1.000			
ANG_2	<---	Anger	1.236	0.057	21.693	***
ANG_3	<---	Anger	0.905	0.046	19.478	***
ANG_4	<---	Anger	0.852	0.046	18.601	***
*** significant at *p* < 0.001						

**Table A2:** Re-specified model’s unstandardized estimates and standard errors.

			Estimate	S.E.	C.R.	P
Perceived_self_efficacy	<---	Job_Demands	−0.169	0.026	−6.415	***
Enjoyment_teaching	<---	Perceived_self_efficacy	0.196	0.033	5.839	***
Enjoyment_teaching	<---	Job_Demands	−0.086	0.022	−3.946	***
Enjoyment_teaching	<---	Job_resources	0.328	0.028	11.537	***
Happiness	<---	Job_resources	0.130	0.022	6.008	***
Happiness	<---	Enjoyment_teaching	0.226	0.034	6.694	***
SM_3	<---	Job_Demands	1.260	0.060	21.108	***
OHQ_9_rev	<---	Happiness	1.000			
OHQ_8	<---	Happiness	1.203	0.191	6.314	***
OHQ_7	<---	Happiness	1.234	0.199	6.187	***
OHQ_6	<---	Happiness	1.323	0.181	7.292	***
OHQ_5	<---	Happiness	2.535	0.314	8.074	***
OHQ_4_rev	<---	Happiness	1.139	0.185	6.157	***
OHQ_3	<---	Happiness	2.671	0.329	8.121	***
OHQ_2	<---	Happiness	2.373	0.295	8.045	***
OHQ_1_rev	<---	Happiness	1.369	0.204	6.722	***
JOY_4	<---	Enjoyment_teaching	1.000			
JOY_3	<---	Enjoyment_teaching	1.109	0.044	24.991	***
JOY_2	<---	Enjoyment_teaching	1.000	0.040	24.729	***
JOY_1	<---	Enjoyment_teaching	0.668	0.033	20.064	***
RLM10	<---	Job_resources	1.000			
RLM9	<---	Job_resources	1.118	0.056	19.944	***
RLM6	<---	Job_resources	0.548	0.056	9.840	***
RLM1	<---	Job_resources	0.852	0.053	16.208	***
SM_5	<---	Job_Demands	0.780	0.059	13.242	***
SM_4	<---	Job_Demands	0.879	0.062	14.120	***
SM_2	<---	Job_Demands	0.974	0.061	16.061	***
SM_1	<---	Job_Demands	1.000			
SM_1A	<---	Perceived_self_efficacy	1.000			
SM_2A	<---	Perceived_self_efficacy	1.050	0.052	20.009	***
SM_5A	<---	Perceived_self_efficacy	0.858	0.053	16.197	***
SM_3A	<---	Perceived_self_efficacy	1.086	0.052	20.895	***
SM_4A	<---	Perceived_self_efficacy	0.885	0.052	16.884	***
RLM5	<---	Job_resources	1.073	0.053	20.160	***
RLM4	<---	Job_resources	1.067	0.055	19.368	***
RLM3	<---	Job_resources	1.187	0.061	19.586	***
RLM2	<---	Job_resources	0.941	0.051	18.520	***
RLM7	<---	Job_resources	0.758	0.047	16.109	***
RLM8	<---	Job_resources	0.967	0.054	17.753	***
*** significant at *p* < 0.001						

## References

[1] Capone V., Petrillo G. (2018). Mental health in teachers: Relationships with job satisfaction, efficacy beliefs, burnout and depression. Curr. Psychol..

[2] Keyes C.L.M. (2007). Promoting and protecting mental health as flourishing: A complementary strategy for improving national mental health. Am. Psychol..

[3] Konu A. (2002). Factor structure of the School Well-being Model. Health Educ. Res..

[4] Diener E. (2009). The Science of Well-Being.

[5] Collie R.J., Shapka J.D., Perry N.E., Martin A.J. (2015). Teacher Well-Being: Exploring Its Components and a Practice-Oriented Scale. J. Psychoeduc. Assess..

[6] Diener E. (2000). Subjective well-being: The science of happiness and a proposal for a national index. Am. Psychol..

[7] Ryan R.M., Deci E.L., Chirkov V.I., Ryan R.M., Sheldon K.M. (2011). A Self-Determination Theory Perspective on Social, Institutional, Cultural, and Economic Supports for Autonomy and Their Importance for Well-Being. Human Autonomy in Cross-Cultural Context.

[8] de la Barrera U., Schoeps K., Gil-Gómez J.-A., Montoya-Castilla I. (2019). Predicting Adolescent Adjustment and Well-Being: The Interplay between Socio-Emotional and Personal Factors. IJERPH.

[9] Kern M.L., Waters L.E., Adler A., White M.A. (2015). A multidimensional approach to measuring well-being in students: Application of the PERMA framework. J. Posit. Psychol..

[10] Michaelson J., Abdallah S., Steuer N., Thompson S., Marks N. (2009). National Accounts of Well-Being: BRINGING Real Wealth onto the Balance Sheet.

[11] Ryff C.D., Singer B. (2000). Interpersonal Flourishing: A Positive Health Agenda for the New Millennium. Pers. Soc. Psychol. Rev..

[12] Wassell E., Dodge R. (2015). A Multidisciplinary Framework for Measuring and Improving Wellbeing. Int. J. Sci. Basic Appl. Res..

[13] Huppert F.A., Cooper C.L. (2014). The State of Wellbeing Science: Concepts, Measures, Interventions, and Policies. Wellbeing.

[14] Ryan R.M., Deci E.L. (2001). On Happiness and Human Potentials: A Review of Research on Hedonic and Eudaimonic Well-Being. Annu. Rev. Psychol..

[15] Keyes C.L.M. (2005). Mental Illness and/or Mental Health? Investigating Axioms of the Complete State Model of Health. J. Consult. Clin. Psychol..

[16] Dodge R., Daly A.P., Huyton J., Sanders L.D. (2012). The challenge of defining wellbeing. Intnl. J. Wellbeing.

[17] Kloep M., Hendry L., Saunders D. A New Perspective on Human Development. https://pdfs.semanticscholar.org/9f30/4cc2ec19dfa4f88c29e6e8c9843511673010.pdf.

[18] Le Cornu R. (2013). Building Early Career Teacher Resilience: The Role of Relationships. AJTE.

[19] Pillay H., Goddard R., Wilss L. (2005). Well-Being, Burnout and Competence: Implications for Teachers. AJTE.

[20] NASUWT (National Association of Schoolmasters Union of Women Teachers) (2017). The Big Question 2017: An Opinion Survey of Teachers and School Leaders.

[21] Lambert R.G., McCarthy C., O’Donnell M., Wang C. (2009). Measuring elementary teacher stress and coping in the classroom: Validity evidence for the Classroom Appraisal of Resources and Demands. Psychol. Schs..

[22] Kilgallon P., Maloney C., Lock G. (2008). Early Childhood Teachers’ Sustainment in the Classroom. AJTE.

[23] Brown L.A., Roloff M.E. (2011). Extra-Role Time, Burnout, and Commitment: The Power of Promises Kept. Bus. Commun. Q..

[24] Hastings R.P., Bham M.S. (2003). The Relationship between Student Behaviour Patterns and Teacher Burnout. Sch. Psychol. Int..

[25] Naghieh A., Montgomery P., Bonell C.P., Thompson M., Aber J.L. (2015). Organisational interventions for improving wellbeing and reducing work-related stress in teachers. Cochrane Database Syst. Rev..

[26] Ross S.W., Romer N., Horner R.H. (2012). Teacher Well-Being and the Implementation of School-Wide Positive Behavior Interventions and Supports. J. Posit. Behav. Interv..

[27] Vesely A.K., Saklofske D.H., Nordstokke D.W. (2014). EI training and pre-service teacher wellbeing. Personal. Individ. Differ..

[28] Manasia L., Ianos M.G., Chicioreanu T.D. (2019). Pre-Service Teacher Preparedness for Fostering Education for Sustainable Development: An Empirical Analysis of Central Dimensions of Teaching Readiness. Sustainability.

[29] Bakker A.B., Demerouti E. (2007). The Job Demands-Resources model: State of the art. J. Manag. Psych..

[30] Skaalvik E.M., Skaalvik S. (2018). Job demands and job resources as predictors of teacher motivation and well-being. Soc. Psychol. Educ..

[31] Cross D. Teacher Wellbeing and Its Impact on Student Learning. www.research.uwa.edu.au/__data/assets/pdf_file/.../teacher-wellbeing-and-student.pdf.

[32] Yin H., Huang S., Wang W. (2016). Work Environment Characteristics and Teacher Well-Being: The Mediation of Emotion Regulation Strategies. IJERPH.

[33] Bermejo-Toro L., Prieto-Ursúa M., Hernández V. (2016). Towards a model of teacher well-being: personal and job resources involved in teacher burnout and engagement. Educ. Psychol..

[34] Collie R.J., Shapka J.D., Perry N.E. (2012). School climate and social–emotional learning: Predicting teacher stress, job satisfaction, and teaching efficacy. J. Educ. Psychol..

[35] Borg M.G., Riding R.J. (1991). Occupational Stress and Satisfaction in Teaching. Br. Educ. Res. J..

[36] Burke R.J., Greenglass E. (1995). A Longitudinal Study of Psychological Burnout in Teachers. Hum. Relat..

[37] Smith M., Bourke S. (1992). Teacher stress: Examining a model based on context, workload, and satisfaction. Teach. Teach. Educ..

[38] Spilt J.L., Koomen H.M.Y., Thijs J.T. (2011). Teacher Wellbeing: The Importance of Teacher–Student Relationships. Educ. Psychol. Rev..

[39] Hamre B.K., Pianta R.C. (2001). Early Teacher-Child Relationships and the Trajectory of Children’s School Outcomes through Eighth Grade. Child Dev..

[40] Newberry M., Davis H.A. (2008). The role of elementary teachers’ conceptions of closeness to students on their differential behaviour in the classroom. Teach. Teach. Educ..

[41] Vera M., Salanova M., Lorente L. (2012). The predicting role of self-efficacyin the Job Demands-Resources Model: A longitudinal study. Estud. de Psicol..

[42] Evans L. (2008). Professionalism, professionality and the development of education professionals. Br. J. Educ. Stud..

[43] OECD (2016). Supporting Teacher Professionalism: Insights from TALIS 2013.

[44] Wang L., Lai M., Lo L.N.-K. (2014). Teacher professionalism under the recent reform of performance pay in Mainland China. Prospects.

[45] Darling-Hammond L., Hyler M.E., Gardner M. (2017). Effective Teacher Professional Development.

[46] Schleicher A. (2018). Valuing Our Teachers and Raising Their Status: How Communities Can Help.

[47] Hendricks M.D. (2014). Does it pay to pay teachers more? Evidence from Texas. J. Public Econ..

[48] Louis K.S. (2006). Changing the Culture of Schools: Professional Community, Organizational Learning, and Trust. J. Sch. Leadersh..

[49] Caprara G.V., Barbaranelli C., Steca P., Malone P.S. (2006). Teachers’ self-efficacy beliefs as determinants of job satisfaction and students’ academic achievement: A study at the school level. J. Sch. Psychol..

[50] Klassen R.M., Chiu M.M. (2010). Effects on teachers’ self-efficacy and job satisfaction: Teacher gender, years of experience, and job stress. J. Educ. Psychol..

[51] Sato T., Haegele J.A. (2018). Physical educators’ engagement in online adapted physical education graduate professional development. Prof. Dev. Educ..

[52] Darling-Hammond L., Flook L., Cook-Harvey C., Barron B., Osher D. (2019). Implications for educational practice of the science of learning and development. Appl. Dev. Sci..

[53] Botke J.A., Jansen P.G.W., Khapova S.N., Tims M. (2018). Work factors influencing the transfer stages of soft skills training: A literature review. Educ. Res. Rev..

[54] García E., Weiss E. (2019). The role of early career supports, continuous professional development, and learning communities in the teacher shortage. The Fifth Report in ‘The Perfect Storm in the Teacher Labor Market’ series.

[55] Ryff C.D. (1989). Happiness is everything, or is it? Explorations on the meaning of psychological well-being. J. Personal. Soc. Psychol..

[56] Chang M.-L. (2009). An Appraisal Perspective of Teacher Burnout: Examining the Emotional Work of Teachers. Educ. Psychol. Rev..

[57] Marcenaro-Gutierrez O.D., Luque M., Lopez-Agudo L.A. (2016). Balancing Teachers’ Math Satisfaction and Other Indicators of the Education System’s Performance. Soc. Indic. Res..

[58] Ross J., Bruce C. (2007). Professional Development Effects on Teacher Efficacy: Results of Randomized Field Trial. J. Educ. Res..

[59] Salanova M., Schaufeli W.B. (2008). A cross-national study of work engagement as a mediator between job resources and proactive behaviour. Int. J. Hum. Resour. Manag..

[60] Danielson C. (2007). Enhancing Professional Practice: A Framework for Teaching.

[61] Hackman J.R. (1980). Work redesign and motivation. Prof. Psychol..

[62] Hackman J.R., Oldham J.R. (1980). Work Redesign.

[63] Ababneh K.I., Hackett R.D. (2019). The direct and indirect impacts of job characteristics on faculty organizational citizenship behavior in the United Arab Emirates (UAE). High Educ..

[64] Bandura A. (1977). Self-efficacy: Toward a unifying theory of behavioral change. Psychol. Rev..

[65] Katsantonis I.G. (2019). Investigation of the Impact of School Climate and Teachers’ Self-Efficacy on Job Satisfaction: A Cross-Cultural Approach. EJIHPE.

[66] Bandura A., Pajares F., Urdan T. (2006). Guide for Constructing Self-Efficacy Scales. Self-Efficacy Beliefs of Adolescents.

[67] Klassen R.M., Tze V.M.C. (2014). Teachers’ self-efficacy, personality, and teaching effectiveness: A meta-analysis. Educ. Res. Rev..

[68] Brouwers A., Tomic W. (2000). A longitudinal study of teacher burnout and perceived self-efficacy in classroom management. Teach. Teach. Educ..

[69] Skaalvik E.M., Skaalvik S. (2011). Teachers’ feeling of belonging, exhaustion, and job satisfaction: The role of school goal structure and value consonance. Anxiety Stress Coping.

[70] Skaalvik E.M., Skaalvik S. (2015). Job Satisfaction, Stress and Coping Strategies in the Teaching Profession—What Do Teachers Say?. IES.

[71] Skaalvik E.M., Skaalvik S. (2007). Dimensions of teacher self-efficacy and relations with strain factors, perceived collective teacher efficacy, and teacher burnout. J. Educ. Psychol..

[72] Lazarus R.S., Folkman S. (2013). Stress, Appraisal, and Coping.

[73] Montgomery C., Rupp A.A. (2005). A Meta-Analysis for Exploring the Diverse Causes and Effects of Stress in Teachers. Can. J. Educ. Rev. Can. de l’éducation.

[74] Chen J. (2016). Understanding teacher emotions: The development of a teacher emotion inventory. Teach. Teach. Educ..

[75] Chen J. (2019). Exploring the impact of teacher emotions on their approaches to teaching: A structural equation modelling approach. Br. J. Educ. Psychol..

[76] Fredrickson B.L., Joiner T. (2002). Positive Emotions Trigger Upward Spirals Toward Emotional Well-Being. Psychol. Sci..

[77] Cohn M.A., Fredrickson B.L., Brown S.L., Mikels J.A., Conway A.M. (2009). Happiness unpacked: Positive emotions increase life satisfaction by building resilience. Emotion.

[78] Fredrickson B.L. (1998). What Good Are Positive Emotions?. Rev. Gen. Psychol..

[79] Fredrickson B.L., Joiner T. (2018). Reflections on Positive Emotions and Upward Spirals. Perspect. Psychol. Sci..

[80] Fredrickson B.L. (2001). The role of positive emotions in positive psychology: The broaden-and-build theory of positive emotions. Am. Psychol..

[81] Fredrickson B.L., Cohn M.A., Coffey K.A., Pek J., Finkel S.M. (2008). Open hearts build lives: Positive emotions, induced through loving-kindness meditation, build consequential personal resources. J. Personal. Soc. Psychol..

[82] Pekrun R., Goetz T., Titz W., Perry R.P. (2002). Academic Emotions in Students’ Self-Regulated Learning and Achievement: A Program of Qualitative and Quantitative Research. Educ. Psychol..

[83] Ashby F.G., Isen A.M., Turken A.U. (1999). A neuropsychological theory of positive affect and its influence on cognition. Psychol. Rev..

[84] Hidi S., Renninger K.A. (2006). The Four-Phase Model of Interest Development. Educ. Psychol..

[85] Frenzel A.C., Pekrun R., Goetz T., Daniels L.M., Durksen T.L., Becker-Kurz B., Klassen R.M. (2016). Measuring Teachers’ enjoyment, anger, and anxiety: The Teacher Emotions Scales (TES). Contemp. Educ. Psychol..

[86] Moors A., Ellsworth P.C., Scherer K.R., Frijda N.H. (2013). Appraisal Theories of Emotion: State of the Art and Future Development. Emot. Rev..

[87] Yao L., Gao J., Chen C., Mu D. (2019). How Does Emotional Labor Impact Employees’ Perceptions of Well-Being? Examining the Mediating Role of Emotional Disorder. Sustainability.

[88] Hills P., Argyle M. (2002). The Oxford Happiness Questionnaire: A compact scale for the measurement of psychological well-being. Personal. Individ. Differ..

[89] Hendres D. (2004). Primary Mental Health Prevention through the Increase of Subjective Well-Being Using Psychotherapeutic Common Factors: A Project Sketch and Preliminary Results. Psychol. Health.

[90] Balgiu B.A. (2018). Psychometric Properties of Curiosity and Exploration Inventory-II (CEI-II) in the Case of A Sample of Romanian Students. Rev. de Psihol..

[91] Joseph G., Daire H., Michael M. (2008). Structural Equation Modelling: Guidelines for Determining Model Fit. Electron. J. Bus. Res. Methods.

[92] MacCallum R.C., Browne M.W., Sugawara H.M. (1996). Power analysis and determination of sample size for covariance structure modeling. Psychol. Methods.

[93] Steiger J.H. (2007). Understanding the limitations of global fit assessment in structural equation modeling. Personal. Individ. Differ..

[94] Tacq J. (1998). Multivariate Analysis Techniques in Social Science Research: From Problem to Analysis.

[95] Prieto L., Salanova M., Martínez I., Schaufeli W. (2008). Extension of the Job Demands-Resources model in the prediction of burnout and engagement among teachers over time. Psicothema.

[96] Buonomo I., Fiorilli C., Benevene P. (2019). The Impact of Emotions and Hedonic Balance on Teachers’ Self-Efficacy: Testing the Bouncing Back Effect of Positive Emotions. Front. Psychol..

[97] Gloria C.T., Faulk K.E., Steinhardt M.A. (2013). Positive affectivity predicts successful and unsuccessful adaptation to stress. Motiv. Emot..

[98] Llorens S., Schaufeli W., Bakker A., Salanova M. (2007). Does a positive gain spiral of resources, efficacy beliefs and engagement exist?. Comput. Hum. Behav..

[99] Wermke W., Olason Rick S., Salokangas M. (2019). Decision-making and control: Perceived autonomy of teachers in Germany and Sweden. J. Curric. Stud..

[100] Maxwell S.E., Cole D.A., Mitchell M.A. (2011). Bias in Cross-Sectional Analyses of Longitudinal Mediation: Partial and Complete Mediation Under an Autoregressive Model. Multivar. Behav. Res..

[101] Goetz T., Becker E.S., Bieg M., Keller M.M., Frenzel A.C., Hall N.C. (2015). The Glass Half Empty: How Emotional Exhaustion Affects the State-Trait Discrepancy in Self-Reports of Teaching Emotions. PLoS ONE.

[102] Global Education Meeting 2018. https://en.unesco.org/themes/education/globaleducationmeeting2018.

